# Congestive Fever

**Published:** 1850-11

**Authors:** E. S. Cooper


### ARTICLE II.

*Congestive Fever.*—Part of a paper read before the Peoria City Medical Society, by E. S. Cooper, M. D.

I shall attempt to portray a disease known more or less familiarly to all practitioners of the West and South, by the various local and general names of Congestive Chill, Malignant Fever, Pernicious Fever, Congestive Fever, &c., &c., all of which are more or less vaguely applied,—particularly the "Congestive Chill," which has of late obtained such gloomy notoriety in the category of Western maladies, and swelled the bills of mortality until the irregular practitioner finds a good excuse for the death of his patient under that specious name. Since the Fall of '38, particularly, has this disease, or the name at least, become the watchword of alarm in many parts of Illinois. To say that this name has been misapplied, would be but speaking the truth, but to say that there is no disease of peculiar fatality attended and particularly characterized by internal venous congestions, would be far from the truth.

Compatibly with my views, a correct nosological arrangement would cut off all the above names, as applied to any Fevers which I have seen, excepting the name of Congestive Fever, which, I think, might with propriety be retained.

The name, in my opinion, is not only pertinent when referring to a character of disease which has come under my observation, but particularly well applied. I shall, therefore, assume that it is a proper term, and endeavor, as I pass along, to prove that it is so.

In describing the disease as it has occurred in my practice, I shall, for the facility of description, adopt a different arrangement from authors who have written on the subject, which arrangement I think indispensable to a correct delineation of its character.

I shall divide it into three types and three varieties.

*Type.*—The type may be Remittent, Intermittent, or Continued.

*Variety.*—The variety may be either Mild, Intervenient, or Malignant.

The importance of this arrangement consists partly in the fact that the first variety is the mildest form of Fever incidental to Illinois, and the latter decidedly the most malignant.

The mild variety is generally of the continued type, and is characterized by more or less diminution in the temperature of the surface; the pulse is a little smaller than natural.

These symptoms continue a longer or shorter period, and pass off spontaneously, by the action of a mild cathartic, or run into one of the other varieties, by exposure to atmospheric vicissitudes, by too great physical and mental exertion, or by the imprudent indulgence of the appetite. Particularly the eating ot indigestible articles of food has been a common cause of change from the Mild Continued variety to the Malignant, Intermittent Paroxysm, which in many instances terminates rapidly fatally, despite the most skilful appliances of our art.

It sometimes occurs that the Mild or Continued variety continues to increase in the severity of the symptoms, from the commencement of the attack until the patient becomes greatly prostrate, without changing its type, but, in my practice, these cases have been rare.

In addition to the causes assigned for the unfavorable change of type, the action of drastic cathartics may convert the mild into fatal symptoms. Of this I shall have further occasion to treat again.

The following case shows the agency which indigestible substances taken into the stomach may have in changing the type, and thereby jeopardizing the life of the patient.

Case.—Mr. A. B.—wagoner by trade—ate freely of cold ham and other meats on the 19th of Nov., 1848, after having walked about for several days, with a cool skin and small pulse. Soon after eating, he was taken with vomiting and purging, and when I saw him, which was about an hour after, he was extremely prostrate, with a cold damp skin and blanched conjunctiva. He vomited up the meats. After recovering from this paroxysm he had an intermission, during which he felt comparatively well.

Two days after the first chill, he had a slight shade of the same, the severity of the symptoms were doubtless prevented by the free use of tonics, during the intermission.

In this case the symptoms might have continued for several days as they had been previously to his taking the meats, or might have slowly declined, without developing anything very important in the character of the disease, but for his having taken the indigestible substances which caused a centripetal direction of the blood.

*Intermittent.*—The Intermittent is regular in the recurrence of its paroxysms, which are Quotidian, Tertian, or Quartan—

though generally of the Tertian grade. It is sometimes fatal in the first paroxysm, frequently in the second, and if not modified by remedial measures, generally in the third. Perhaps no disease, incidental to the West, affords the same field for the triumphs of our art. The worst cases, if treated vigorously from the beginning, are almost invariably amenable to medicine, but so far as I am prepared to say, almost all die when left to themselves. The following case shows, particularly, the fatal tendency of the disease when the aid of medicine is not sought for in the early stages, and is precisely like many others which have occurrèd in the practice of every medical man in the sections where the disease prevails.

Case.—Mr. S., æt. 60, resident of Tazewell Co., near Drew's Mill, was attacked on the 21st of Aug., 1848, with a chill which prostrated him for some hours, and produced considerable subsequent debility, though he was able to walk about the house next day. On the subsequent day which was the 23d, I was called, and found him in the following condition: Pulse almost imperceptible; skin cold, much relaxed, and covered with large drops of cold sweat; lips and cheeks of a purple hue, as were, also, many parts of the surface, especially the hands and feet; conjunctiva blanched; eyes glassy and suffused with tears; countenance idiotic; tongue pale and tremulous; breathing laborious. The patient had been vomiting in the commencement, which was three hours previously. The chill lasted two hours longer, and terminated fatally.

*Remittant.*—The Remittent is much more mild than the Intermittent, though I think it sometimes runs into that type, and the patient dies with all the symptoms characteristic of that frightful malady popularly known among us as the "Congestive Chill."

This type has occurred less frequently in my practice than either of the others. Sinking of the pulse, greater or less in proportion to the severity of the attack, I consider indispensable to constitute a Congestive Fever, and in the Remittent form of this disease, the remission is characterized by an increase of fullness, by the temperature of the surface rising. This form of the disease is comparatively mild, and unless by its running into the Intermittent, patients are seldom lost, though I saw it prevail in the eastern part of Illinois, where the disease was not subdued short of from five to ten days, medication. *Continued.* The continued form of Congestive, is the mildest form of febrile affection with which I have been acquainted and is characterized by a slight reduction in the temperature of the surface, the pulse is a little smaller than natural, with a general feeling of torpor, with uneasy sensations in the region of the stomach; the countenance is dejected.

These symptoms continue a longer or shorter period, often without prostrating very much, and finally subside without any efforts but the powers of nature. More frequently it requires a mild aperient, without which the disease very often changes the type and assumes a character very different and extremely difficult to treat successfully.

*Pathology.*—General internal venous congestion is the prominent feature in the pathology of most of these cases. In many instances some particular part is the principal seat of congestion, while the veins of other parts are slightly engorged.

The cavæ, with other vessels about the heart, may be the principal seat of engorgement, or the lungs, or the brain, and spinal cord, or all of these may be simultaneously engorged, as is the case in the fatal attacks of the disease. In the worst attacks the engorgement of the lungs constitutes the most prominent symptom, and in these cases the patient is borne down with a mysterious rapidity very like the worst attacks of Cholera. The lungs alone may be much engorged, and produce the most frightful and rapid collapse without an evacuation, except by sweating.

The accompanying symptoms indicate positively the pathological condition of the parts diseased, when the lungs are the principal seat of Venous Congestion. Thus the purple lips and cheek, the blue skin accompanied with the cold extremities and extremely small pulse, laborious breathing, are not to be mistaken. As I can illustrate what appears to me to be the true pathology of the diseases in these attacks more clearly by introducing a case just here, I beg leave to do it:

*Case.*—Mr. W. E. R., æt. 43, forwarding and commission merchant, very temperate habits, but rather feeble constitution: attacked on the 9th of July, 1848, at 8 A. M., after having walked to his warehouse just before. At half past eight I saw him and found the following his condition: Pulse almost imperceptible; tongue pale and tremulous; conjunctiva blanched; skin cold, damp and relaxed; lips and cheeks purple, surface generally the same hue.

The patient was unable to turn himself in bed soon after the attack. He recovered from the paroxysm, but had a slight shade of the same symptoms on the second day at the same hour, having in the meantime taken largely of tonics to interrupt the anticipated chill.

In this case there was extreme engorgement in the veins of the lungs, and instead of producing increased vascular and nervous excitement, as is the case when the arteries are engorged, the action is as little if not less than in the natural state. I have even thought sometimes the excitement of a part or parts laboring under venous congestion was diminished in proportion to the extent of the congestion. In extreme congestion of the lungs the bronchial lining becomes relaxed and pours out a muco-serous fluid in the same way that the skin pours out the watery portions of the blood. The fluid effused from the lining membrane of the bronchia besmears that membrane and interrupts the contact of atmosphere with the blood, whereby its decarbonization is prevented, and venous blood being thrown back to the left side of the heart, in this state is carried into the system and acts as a slow narcotic poison.

This, it appears to me, must be the true state of all those cases of extreme "congestive chill," in which the patient is borne down and exhausted without any evacuation but by the skin.

In other cases, where vomiting and purging are early and constant symptoms, the liver and its adjacent veins are greatly congested, the veins of the stomach and mesentery are likewise engorged.

When the veins of the brain are greatly distended the patient has an idiotic countenance, eyes glassy, conjunctiva blanched, complete torpor of the sensorial powers, extreme prostration of physical strength, and delirium. But no increase of heat about the head attends these cases. The skin of the forehead participates in the general dampness and relaxation which is common to the surface.

Whether the nervous system is the primary seat of derangement, as is contended by many, the accompanying symptoms are evidently the result of congestions in the venous system.

If not, why is it that in or during the intermittent paroxysm of the disease in one instance our patient is rapidly reduced by vomiting and purging unaccompanied with any blue tint of the skin. Another is reduced still more rapidly without any evacuation except by sweating, with an extraordinary change in the color of the skin.

What is it that produces this marked difference in the color of the skin in these cases? Could nervous influence alone, or unaccompanied with congestion of the lungs, produce such a want of decarbonization of the blood as is evinced in these latter cases?

If one patient dies with an idiotic countenance, blanched conjunctiva and early delirium; another dies with none of these symptoms, but with vomiting and purging, and yet another dies with all these symptoms combined, and if, in the first case, congestion is found in the veins of the brain and spinal cord upon post mortem inspection; in the second, the lungs; in the third, the liver, stomach, and mesentery, and in the last more or less congestion in all, it appears to me, we can account for the difference in the symptoms by what is found to be the pathological condition of the parts, in the post mortem examination, without puzzling our brain to look beyond, into the mysteries of the nervous system, which at best accounts for but few of the various phenomena. If, then, we have found the pathological state, as evinced by post mortem inspection, to account satisfactorily for all the symptoms, and that pathological state to be internal venous congestion, then I urge the propriety of using the term Congestive Fever, particularly as many of its symptoms are more or less common to other Fevers.

